# Skull evolution and lineage diversification in endemic Malagasy carnivorans

**DOI:** 10.1098/rsos.240538

**Published:** 2024-10-23

**Authors:** Chris J. Law, Tate J. Linden, John J. Flynn

**Affiliations:** ^1^ Burke Museum and Department of Biology, University of Washington, Seattle, WA, USA; ^2^ Division of Paleontology, American Museum of Natural History, New York, NY, USA

**Keywords:** adaptive radiation, Carnivora, craniomandibular, Madagascar, phenotypic evolution, phylogenetic comparative methods

## Abstract

Madagascar is one of the world’s foremost biodiversity hotspots with more than 90% of its species endemic to the island. Malagasy carnivorans are one of only four extant terrestrial mammalian clades endemic to Madagascar. Although there are only eight extant species, these carnivorans exhibit remarkable phenotypic and ecological diversity that is often hypothesized to have diversified through an adaptive radiation. Here, we investigated the evolution of skull diversity in Malagasy carnivorans and tested if they exhibited characteristics of convergence and an adaptive radiation. We found that their skull disparity exceeds that of any other feliform family, as their skulls vary widely and strikingly capture a large amount of the morphological variation found across all feliforms. We also found evidence of shared adaptive zones in cranial shape between euplerid subclades and felids, herpestids and viverrids. Lastly, contrary to predictions of adaptive radiation, we found that Malagasy carnivorans do not exhibit rapid lineage diversification and only marginally faster rates of mandibular shape evolution and to a lesser extent cranial shape evolution, compared to other feliforms. These results reveal that exceptional diversification rates are not necessary to generate the striking phenotypic diversity that evolved in carnivorans after their dispersal to and isolation on Madagascar.

## 1. Introduction

Many islands are considered as biodiversity hotspots [1] and contain species that are not found anywhere else in the world [2–4]. High rates of endemism could be spurred by adaptive radiations [5]. Islands in particular, which are often geographically isolated, provide novel ecological opportunities for founding species to rapidly diversify to a variety of species and phenotypes to fill previously unoccupied ecological niches [6–8]. Unsurprisingly, the best-studied adaptive radiations are found among insular island groups such as Galápagos finches, Hawaiian honeycreepers and Caribbean anoles [9–11]. One of the world’s foremost biodiversity hotspots is Madagascar, with more than 90% of its species endemic to the island [12]. As Madagascar has been geographically isolated for over 88 million years, most endemic Malagasy vertebrates are a result of several independent successful dispersal events from Africa or Asia [13]. Upon arrival on Madagascar, adaptive radiations are hypothesized to have facilitated the diversification of many Malagasy clades, such as beetles [14,15], frogs [16,17], vangas [18] and lemurs [19–21]. In this study, we investigate the diversity of Malagasy euplerid carnivorans, one of four extant terrestrial mammalian clades (i.e. Lemuroidea, Eupleridae, Tenrecidae and Nesomyinae) endemic to Madagascar and hypothesized to have diversified through an adaptive radiation [22].

**Figure 1.**
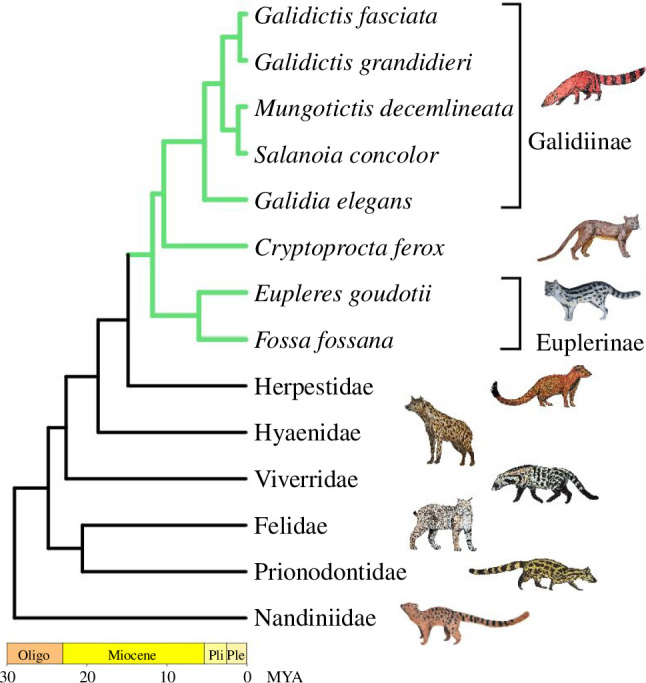
Phylogeny of Eupleridae and other extant feliform Carnivora families. Phylogeny based on [33]. Oligo, Oligocene; Ple, Pleistocene; Pli, Pliocene.

Although there are only eight extant species, Malagasy carnivorans exhibit remarkable phenotypic and ecological diversity [22–25], so much so that no anatomical character can be used to define them. As a result, Malagasy carnivorans have traditionally been thought to belong to or originate from multiple feliform families (i.e. Herpestidae, Viverridae and Felidae) and thus presumably dispersed to Madagascar through multiple independent dispersal events [26–29]. Molecular data have since revealed that all Malagasy carnivorans originated from a single ancestor, thus forming a monophyletic clade and most likely exhibited a single dispersal event to Madagascar [30–32]. Yoder *et al.* [30] assessed the four potential models that could explain the appearance(s) of the euplerid ancestor on Madagascar: Gondwanan vicariance; landbridge connections and ‘sweepstakes’ over-water dispersal in a single event (i.e. ‘rafting’) or in multiple steps via island hopping. They concluded that available data are most consistent with the common ancestor of a monophyletic Eupleridae arriving in Madagascar ~24–18 million years ago via a single over-water dispersal from African ancestry. Eupleridae consists of three major clades: Galidiinae, Euplerinae and the fossa (*Cryptoprocta ferox*) (figure 1). Galidiines are comprised of five species of vontsira. Their superficial resemblance to mongooses (Herpestidae) has led to their ‘Malagasy mongoose’ alias and even to their classification as herpestids [26,27]. Galidiines exhibit diverse ecologies such as in diets that range from insectivory, similarly found in herpestids, to more carnivorous diets in *Galidictis* species [22]. Euplerines superficially resemble civets (Viverridae), leading to them having been described as ‘Malagasy civets’ and classified as viverrids [28]. Similar to viverrids, euplerines exhibit diverse ecologies ranging from the omnivorous fanaloka (*Fossa fossana*) that feeds on a variety of invertebrate and vertebrate prey to the eastern falanouc (*Eupleres goudotii*) that specialize on soft-bodied invertebrates such as worms and slugs [22]. The last euplerid clade consists of the fossa, the largest and most carnivorous of the extant euplerids [34]. Its large-bodied civet-like and cat-like resemblances have historically led its placement within either Viverridae or Felidae [28,29].

Here, we investigated the morphological disparity among Malagasy euplerids and in comparison to taxa in other feliform carnivoran groups with which they have been historically classified. We used skull shape in our examination of phenotypic evolution because of its strong associations with dietary ecology across carnivorans [35–38]. Because of their superficial resemblances, we also investigated convergence among euplerid and feliform clades with which they have been classified historically. We predict that euplerids will exhibit greater disparity compared to other feliform clades and that the three euplerid clades—galidiines, euplerines and the fossa—will converge towards herpestid-, viverrid- and felid-like morphotypes in skull morphology, respectively. The observed phenotypic and ecological diversities found in euplerids suggest that they exhibited an adaptive radiation. Therefore, we also test if rates of their lineage diversification and phenotypic evolution are significantly accelerated relative to other clades as predicted under adaptive radiation. We predict that euplerids will exhibit shifts towards faster rates of lineage diversification and phenotypic evolution compared to other feliform clades.

## 2. Methods

### 2.1. Morphological data

We quantified the shapes of 110 crania across 57 feliforms (~50% of species diversity) and the shapes of 315 mandibles across 90 feliforms (~79% of species diversity) using three-dimensional (3D) geometric morphometrics [39,40]. Our dataset encompasses all eight euplerid species (number of specimens per species ranged from 1 to 5). Whether *Eupleres major* and *Salanoia durrelli* are true species remains debated [41,42]. Shape data were collected from 3D scans of feliform skulls obtained from surface scanning with an EinScan Pro HD scanner at various museums, an Artec Spider structured light 3D scanner at the American Museum of Natural History, or from previously published work [36] (see electronic supplementary material, table S1, for list of specimens and museums). All specimens were fully mature, determined by the closure of exoccipital–basioccipital and basisphenoid–basioccipital sutures on the cranium and full tooth eruption. Although carnivorans exhibit sexual dimorphism [43,44], a combination of females, males and sex unknown individuals was used because just one sex cannot be used without compromising the inclusion of as many euplerid species as possible. We used 35 landmarks and seven curves with 134 semi-landmarks for the cranium and 21 landmarks and four curves with 24 semi-landmarks for the mandible (electronic supplementary material, figure S1). Landmarks were digitized using Slicer, and curves were digitized by oversampling semi-landmarks in Slicer. Landmarks were superimposed by generalized Procrustes analysis, and semi-landmarks on the curves were allowed to slide along their tangent vectors until their positions minimized bending energy [40,45]. As part of the superimposition procedure, bilaterally homologous landmarks and semi-landmarks were reflected across the median plane and averaged using the geomorphic function bilat.symmetry. All Procrustes superimpositions were performed in the R package geomorph v. 4.0.6 [46]. We used centroid size as our metric of cranial and mandibular size.

### 2.2. Craniomandibular shape analyses

We performed all analyses under a phylogenetic framework using a phylogeny of mammals generated by Upham *et al*. [33] and pruned to include only feliform carnivorans. All analyses were performed in R v. 4.1.1 [47]. Because allometry has been shown to facilitate or constrain skull shape evolution [48], we first tested for evolutionary allometry on cranial and mandibular shape by performing a phylogenetic Procrustes regression [49] with a random residual permutation procedure (1000 iterations) using the geomorph function procD.pgls. Both cranial shape (sum of squares (SS) = 0.022, mean squares (MS) = 0.022, *R*^2^ = 0.20, *F* = 14.127, *Z* = 3.45, *p* < 0.001) and mandibular shape (SS = 0.044, MS = 0.044, *R*^2^ = 0.25, *F* = 29.360, *Z* = 5.07, *p* = 0.001) exhibited significant evolutionary allometry. We, therefore, extracted allometry-free shape as the shape residuals from the phylogenetic Procrustes regressions and used them in all subsequent analyses.

#### 2.2.1. Craniomandibular shape disparity

We visualized the phylomorphospace of cranial and mandibular shape by performing principal component analyses (PCAs) using the geomorph function gm.prcomp. We then examined if cranial and mandibular disparity (i.e. Procrustes variance) differed among the feliform clades using the geomorph functions procD.plgs and morphol.disparity.

#### 2.2.2. Testing for convergence towards different feliform morphotypes

Euplerids are often cited to superficially resemble and were historically classified together with other feliforms, specifically galidiines with mongooses (Herpestidae), euplerines with civets (Viverridae) and the fossa with cats (Felidae) or with civets. Therefore, we tested whether there was statistical evidence for convergence in these various euplerid–feliform comparisons. Here, we define convergence as lineages evolving to be more similar to one another than were their ancestors [50–52]. We assessed convergence using the *Ct* measure in the R package convevol [51,52], which tests whether the geometric distance between focal lineages in phylomorphospace is shorter than the distances between ancestral nodes at a specific time point. We then used a frequency-based measure of convergence (*C*_5_), which determines whether the number of lineages that crosses into a particular region of morphospace is greater than expected. We conducted 100 simulations under Brownian motion to assess statistical significance of *Ct* and *C*_5_ measures. Together, these measures provide a more complete test of our definition of convergence. We also applied the *θ*_real_ measurement in the search.conv in the R package RRphylo [53]. *θ*_real_ is the angle between the phenotypic vectors of focal lineages where smaller angles indicate greater phenotypic similarity, which in turn may reflect convergence of taxa towards a common morphology [53]. A caveat of this approach is that it does not distinguish convergence from conservatism.

Lastly, we tested whether each of the three groups exhibited shared adaptive zones in skull shapes by fitting five multivariate evolutionary models [54,55] on the first three principal components (PCs) of the cranial shape dataset (accounting for 74.44% of total cranial shape variation) and mandibular shape dataset (87.4% of total mandibular shape variation) using the R package mvMORPH v. 1.1.7 [56] to incorporate covariances between axes. These models included single-rate Brownian motion model (mvBM1), single-peak Ornstein–Uhlenbeck model (mvOU1) and three two-peak Ornstein–Uhlenbeck models (mvOUM). Each of these mvOUM models designated two optima, one optimum for the focal comparison and a second optimum for the remaining feliforms. All models were fitted across 250 mapped trees to account for uncertainty in phylogenetic topology and stochastic character maps of group designations. Models were assessed with small sample corrected Akaike weights (AICcW), and all models with a ∆AICc < 2 were considered equally best-fitting models. Support for a mvOUM model as a best-fitting model would suggest a shared adaptive zone between the focal clades of interest. These results, however, also would provide more nuanced interpretations of the convergent evolution because they are unable to distinguish between potential convergence or conservatism [51,52].

#### 2.2.3. Craniomandibular shape evolutionary rates

We investigated rates of cranial shape and mandibular shape evolution using two approaches. First, we tested if evolutionary rates in cranial shape and mandibular shape differed among the feliform clades using the geomorph function compare.evol.rates. Second, we examined branch-specific evolutionary rates and rate shifts of cranial shape and mandibular shape using a variable rates model under a reversible-jump Markov chain Monte Carlo (rjMCMC) framework in BayesTraits v4.1.1 (www.evolution.reading.ac.uk). To reduce the dimensions of the shape data for BayesTraits analysis, we used only the phylogenetic principal component scores (pPCs) that represent >95% of the shape variation in the cranium (first 18 pPCs) and mandible (first 11 pPCs). We ran two independent chains, each with 200 000 000 iterations sampled every 20 000 iterations. After the first 25 000 000 iterations were discarded as burn-in, we assessed convergence of the two chains by checking the trace plots and then the effective sample size with Gelman and Rubin’s diagnostics in the R package coda v. 0.19-4. We plotted rate shifts and branch-specific rates across the feliform phylogeny and constructed density plots to compare evolutionary rates among feliform families using custom functions from [57]. A caveat to these analyses is that, outside of Eupleridae, we have more limited representation of ~50% and ~79% of species diversity of the remaining feliform clades in our cranial and mandibular shape datasets, respectively. Nevertheless, although identification of significant rate shifts potentially could be influenced by sampling in those other feliform clades, they are not the focus of this study.

### 2.3. Rates of lineage diversification

We estimated speciation and extinction rates through time and across the feliform phylogeny using two methods, Bayesian analysis of macroevolutionary mixtures (BAMM) [58] and the lineage-specific birth–death–shift (LSBDS) model [59]. BAMM applies a rjMCMC to explore candidate models of lineage diversification as well as quantify heterogeneity in evolutionary rates. We performed two independent BAMM runs of 5 million generations on feliform phylogeny, sampling every 1000 generations and with priors chosen using the R package BAMMtools v2.0 [60]. We assessed the convergence of each BAMM run using the R package BAMMtools v. 2.1.11 [60]. The LSBDS model samples rate regimes from a prior distribution, discretized into a fixed number of rate categories. We implemented the LSBDS model using seven categories (one for each of the seven extant feliform families) for speciation and extinction and ran two MCMC chains for 5000 generations in RevBayes [61]. We merged the posteriors, retaining the last 4000 generations from the MCMC in the R package RevGadgets [62]. Convergence for both methods was assessed by checking that the ESS values for all model parameters in the log files were greater than 200, using the R package coda [63].

## 3. Results

### 3.1. Craniomandibular shape morphospace and disparity

**Figure 2.**
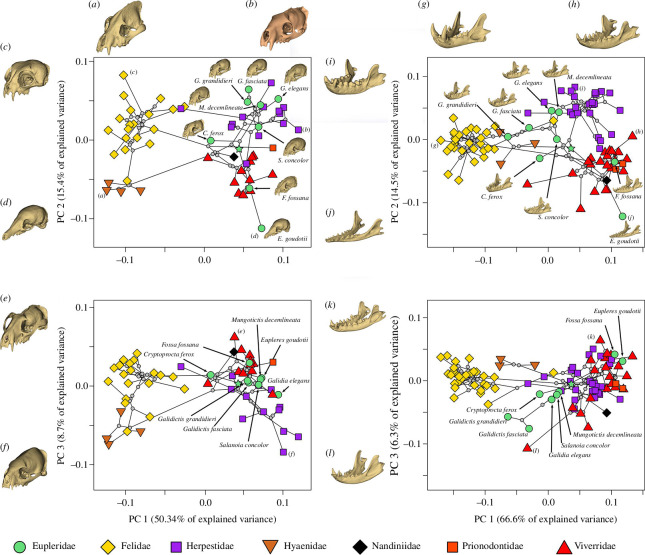
Morphospace of allometry-free cranial (left) and mandibular (right) shape defined by PC axes 1−3. Taxa illustrated for the cranial morphospace: (*a*) bBrown hyena (*Hyaena brunnea* (Hyaenidae)), –PC 1; (*b*) Javan mongoose (*Urva javanicus* (Herpestidae)), +PC 1; (*c*) Pallas’s cat (*Otocolobus manul* (Felidae)), +PC 2; (*d*) eastern falanouc (*E. goudotii* (Eupleridae)), –PC 2; (*e*) Asian palm civet (*Paradoxurus hermaphroditus* (Eupleridae)), +PC 3; and (*f*) Egyptian mongoose (*Herpestes ichneumon* (Herpestidae)), –PC 3. Taxa illustrated for the mandibular morphospace: (*g*) tiger (*Panthera tigris* (Felidae)), –PC 1; (*h*) small Indian civet (*Viverricula indica* (Viverridae)), +PC 1; (*i*) Ethiopian dwarf mongoose (*Helogale hirtula* (Herpestidae)), +PC 2; (*j*) eastern falanouc (*E. goudotii* (Eupleridae)), –PC 2; (*k*) large Indian civet (*Viverra zibetha* (Viverridae)), +PC 3; and (*l*) binturong (*Arctictis binturong* (Viverridae)), –PC 3. Grey circles indicate estimated ancestral shapes at nodes, and the green star indicates estimated ancestral shape of the ancestral euplerid node.

**Figure 3.**
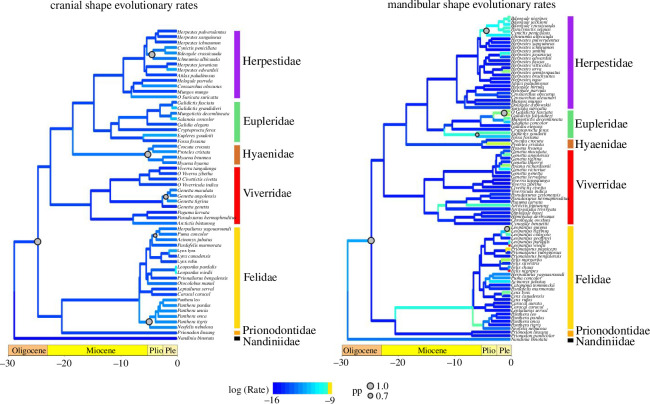
Phylorate plots of cranial and mandibular shape evolutionary rates across feliform Carnivora. Warmer colours indicate faster rates and cooler colours indicate slower rates. Significant shifts in evolutionary rate are shown as grey circles with sizes proportional to the posterior probability. All rates were log-transformed.

PCs 1−3 explain 74.44% of the cranial shape variation across feliforms (figure 2). PC1 distinguishes hypercarnivorous felids and hyaenids (negative PC1) from the remaining, less carnivorous feliforms including euplerids (positive PC1). Positive PC1 is characterized by lateral narrowing of the cranium at the zygomatic arches, expansion of the braincase through widening of the nuchal crests and reduction of the sagittal crest resulting in dorsoventral flattening. The diversity of euplerid cranial shapes is best captured by PC2, as euplerid species occupy the full range of PC2. Positive PC2 describes shortening of the rostrum and broadening of the cranium at the zygomatic arches and nuchal crests. Galidiines are associated with positive PC2 and are encompassed within the range of variation of herpestids (and felids), whereas euplerines are associated with negative PC2 with *F. fossana* falling within the range of viverrids (and hyaenids) but *E. goudotii* distinct from all other feliforms. In PC 2, *Cryptoprocta* is intermediate between galidiines and euplerines (closer to the former) and falls within the broad range of values of felids. In the total PC1/2 cranial morphospace, galidiines cluster with herpestids, euplerines are closest to and overlap with viverrids and *Cryptoprocta* is distinct from other feliforms. Euplerids show little variation along cranial PC3, falling entirely within the range of felids and overlapping with the lower range of viverrids and upper range of herpestids. All other non-monotypic feliform families show a greater range of variance than and/or do not overlap with euplerids in PC3. Positive PC3 describes slight elongation and ventrodorsal flexion of the rostrum and slight broadening of the zygomatic arches. Ancestral node reconstruction suggests that euplerids exhibit a cranial shape between viverrids and herpestids (figure 3). Overall, euplerids exhibit significantly greater cranial shape disparity (Procrustes variance = 0.0078) compared to felids (0.0047, *p* = 0.026) and viverrids (0.0033, *p* = 0.004), but not hyaenids (0.0066, *p* = 0.595) or herpestids (0.0061, *p* = 0.299).

Euplerids occupy greater regions of mandibular morphospace when compared to cranial morphospace. PCs 1−3 explain 87.4% of the mandibular shape variation across feliforms (figure 2). Like in the crania, PC1 of mandibular shape largely distinguishes hypercarnivorous felids and to a lesser extent hyaenids (both with negative PC1 values), from the remaining, less carnivorous feliforms (positive PC1), except for overlap with hyaenids of two species of *Galidictis* (galidiine euplerids) and one herpestid. In mandibular PC 1, euplerines fall within the range of viverrids and a few herpestids at the extreme positive end of their range; galidiines have a wide range of mandibular PC1 values, overlapping with herpestids at the most negative end of their range of variation, hyaenids, and one species each of felid and viverrid; *Cryptoprocta* is very close to hyaenids in PC1 value. Positive PC1 describes dorsoventral mandibular flexure and elongation, decrease in coronoid height and lateral compression of the mandibular body. However, unlike in the crania, euplerids exhibit greater variation along PC1 than in any other feliform family (although viverrids are similar in range of variation, due to one extreme outlier, the binturong (*A. binturong*)) and span negative and positive PC1.

Euplerids also approach both the negative and positive ends of variation in both PCs 2 and 3, with *E. goudotii* again having the most extreme negative value for PC 2 of any feliform (as for the cranial PCA). Positive PC2 describes anteroposterior shortening of the mandibular body, increases in coronoid height and lateral broadening of the mandibular body; positive PC3 describes anteroposterior elongation of the mandibular body, dorsoventral compression of the mandibular body and reduction in coronoid height. Ancestral node reconstruction suggests that euplerids exhibit a cranial shape between viverrids and herpestids, with closer resemblance to the latter (figure 3). Euplerids exhibit significantly greater mandibular shape disparity (Procrustes variance = 0.0100) compared to felids (0.0035, *p* = 0.001), viverrids (0.0054, *p* = 0.020) and herpestids (0.0040, *p* = 0.003) but not hyaenids (0.0050, *p* = 0.692).

### 3.2. Convergence towards different feliform morphotypes

We did not find statistical support for the distance-based measure of convergence between galidiines and herpestids (cranial *Ct*_1_ = −0.19, *p* = 0.96; mandibular *Ct*_1_ = −0.03, *p* = 0.39), between euplerines and viverrids (cranial *Ct*_1_ = −0.24, *p* = 0.84; mandibular *Ct*_1_ = −0.25, *p* = 0.84), between the fossa (*C. ferox*) and felids (cranial *Ct*_1_ = 0.05, *p* = 0.08; mandibular *Ct*_1_ = −0.05, *p* = 0.48) or between the fossa and viverrids (cranial *Ct*_1_ = −0.31, *p* = 0.98; mandibular *Ct*_1_ = −0.17, *p* = 0.46). The frequency-based measure of convergence indicated that the number of transitions into regions of phylomorphospace occupied by herpestids, viverrids, and felids was not significantly greater than expected under Brownian motion (all *p* > 0.380). Similarly, we did not find statistical support for smaller *θ*_real_ angles between galidiines and herpestids (cranial *θ*_real_ = 7.42, *p* = 0.051; mandibular *θ*_real_ = 8.52, *p* = 0.401), between euplerines and viverrids (cranial *θ*_real_ = 8.71, *p* = 0.645; mandibular *θ*_real_ = 8.90, *p* = 0.747), between the fossa (*C. ferox*) and felids (cranial *θ*_real_ = 8.28, *p* = 0.688; mandibular *θ*_real_ = 8.64, *p* = 0.499) or between the fossa and viverrids (cranial *θ*_real_ = 7.68, *p* = 0.199; mandibular *θ*_real_ = 8.91, *p* = 0.738).

Using evolutionary modelling, we found evidence of shared adaptive zones in cranial shape between galidiines and herpestids (best model = mvOUM, AICcW = 0.98) and some evidence of shared adaptive zones in cranial shape between euplerines and viverrids (best models = mvOUM (AICcW = 0.52) and mvBM (AICcW = 0.47; ∆AICc = 0.20)) and between the fossa and felids (best models = mvBM (AICcW = 0.66) and mvOUM (AICcW = 0.32; ∆AICc = 1.47)) (electronic supplementary material, table S2). In contrast, we found no evidence of shared adaptive zones in mandibular shape in all three comparisons (best model for all = mvOU1 (AICcW > 081); electronic supplementary material, table S2).

### 3.3. Rates of craniomandibular shape evolution

Rates of cranial shape evolution in euplerids (ln *σ*^2^_multi_ = −12.86) do not significantly differ from most feliform clades (felid ln *σ*^2^_multi_ = −12.94, *p* = 0.592; hyaenid ln *σ*^2^_multi_ = −12.47, *p* = 0.100; herpestid ln *σ*^2^_multi_ = −13.14, *p* = 0.105), except for viverrids (ln *σ*^2^_multi_ = −13.72, *p* = 0.001), in which euplerids exhibited significantly faster rates of cranial shape evolution. Although we found six significant rate shifts (posterior probability (pp) > 0.70) across feliforms, none of these occurred within euplerids (figure 3). Instead, rate shifts occurred towards or within the branches of the other major feliform clades including Hyaenidae, Felidae (pantherines and the *Puma* lineage), Viverridae (crownward *Genetta* species), Herpestidae (subclade of herpestines) and the overall feliform clade excluding Nandiniidae (figure 3).

Euplerids exhibited greater rate heterogeneity in evolution of mandibular shape compared to cranial shape. Euplerids exhibited significantly faster rates of mandibular shape evolution (ln *σ*^2^_multi_ = −12.29) compared to viverrids (ln *σ*^2^_multi_ = −13.08, *p* = 0.001) and herpestids (ln *σ*^2^_multi_ = −12.79, *p* = 0.001) but significantly slower rates compared to felids (ln *σ*^2^_multi_ = −11.80, *p* = 0.023). There was no difference in mandibular shape evolutionary rates between euplerids and hyaenids (ln *σ*^2^_multi_ = −12.23, *p* = 0.889). Furthermore, we found that two of the five significant rate shifts in mandibular shape (pp > 0.70) occurred within euplerid clades: *Galidictis* species and euplerines (figure 3). The remaining rate shifts occurred within the same subclade of herpestines that exhibited a rate shift in cranial shape evolution, within *Leopardus* cats, and for the overall feliform clade excluding Nandiniidae (figure 3).

### 3.4. Diversification rate analyses

**Figure 4.**
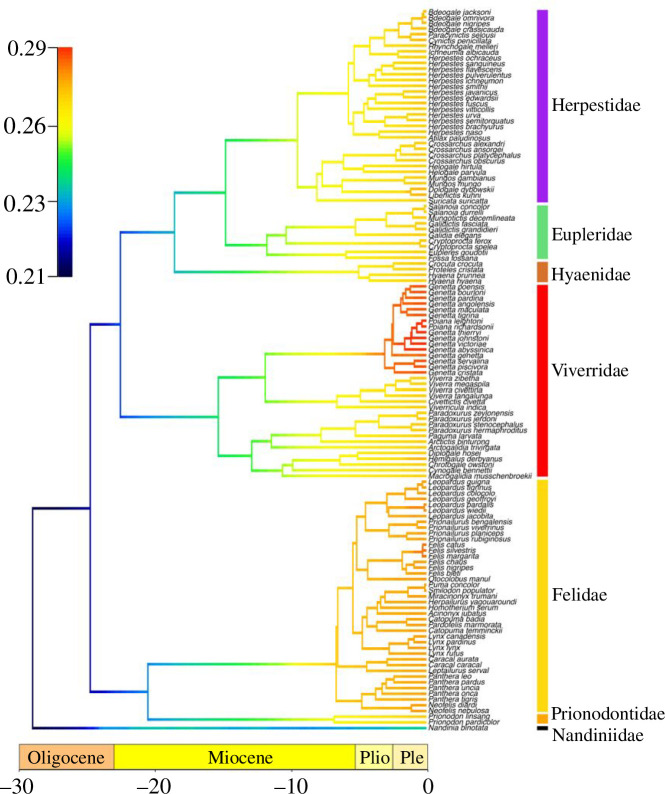
Phylorate plot of lineage diversification rates across feliform Carnivora using BAMM. Colours at each point in time along the branches of the phylorate plot denote instantaneous rate of diversification. Warmer colours indicate faster rates and cooler colours indicate slower rates. There are no significant shifts in diversification rates across the phylogeny. Analyses of lineage diversification rates using LSBDS resulted in similar patterns (electronic supplementary material, figure S2).

Euplerids do not exhibit distinct rates of diversification, as both BAMM and the LSBDS models are united in revealing that there are no increased species diversification rates on the branches leading towards Eupleridae compared to the remaining feliforms (figure 4; electronic supplementary material, figure S2). Furthermore, we found no significant shifts in diversification rate within feliforms, albeit there are slightly elevated rates on the branches leading towards to the viverrid subfamily Genettinae and felids (figure 4; electronic supplementary material, figure S2).

## 4. Discussion

### 4.1. Skull diversity in Malagasy carnivorans

**Figure 5.**
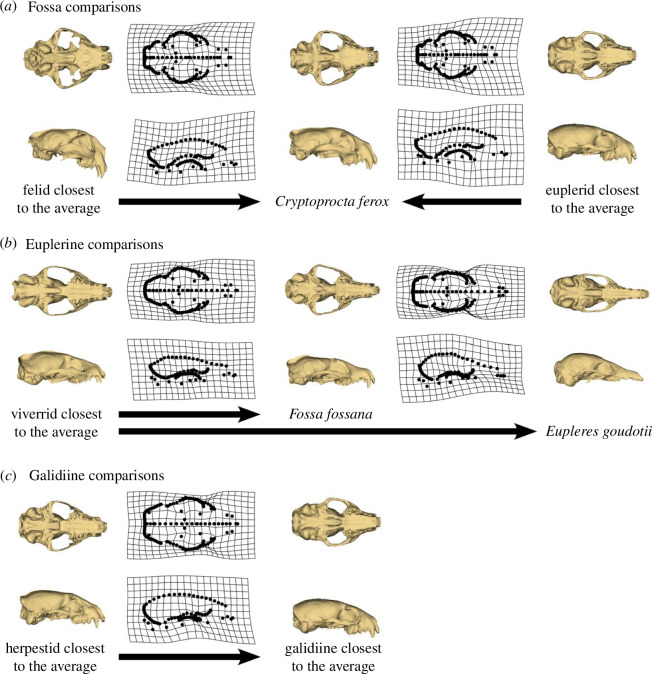
Thin-plate spline deformation grids depicting cranial shape differences between euplerid clades and other feliform families. (*a*) Cranial shape of the fossa (*C. ferox*) appears intermediate between felids and other euplerids. (*b*) Cranial shape of euplerines: the fanaloka (*F. fossana*) resembles viverrids, whereas the eastern falanouc (*E. goudotii*) exhibits a more specialized morphology. (*c*) Cranial shapes of most galidiines resemble herpestids.

**Figure 6.**
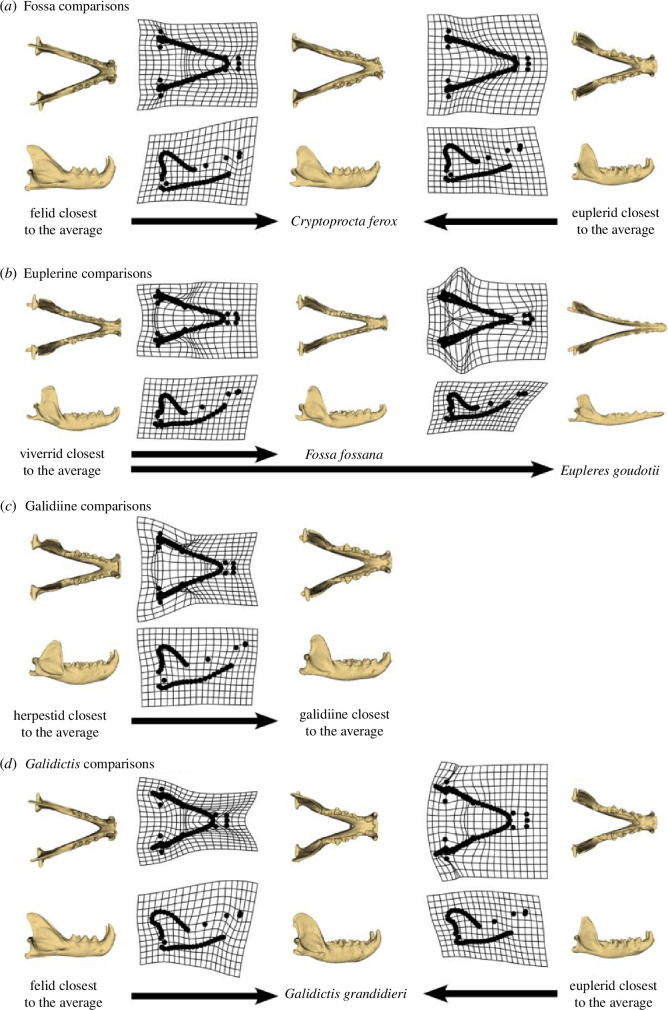
Thin-plate spline deformation grids depicting mandibular shape differences euplerid clades and other feliform families. (*a*) Mandibular shape of the fossa (*C. ferox*) appears intermediate between felids and euplerids. (*b*) Mandibular shape of euplerines: the fanaloka (*F. fossana*) resemble viverrids, whereas the eastern falanouc (*E. goudotii*) exhibits a more specialized morphology. (*c*) Mandibular shapes of most galidiines resemble herpestids, although the mandibular shape (*d*) of Grandidier’s vontsira (*G. grandidieri*) appears intermediate between felids and other galidiines.

Euplerids exhibit greater disparity in their skulls compared to most feliform families, for both cranial and mandibular shapes, and particularly so in their mandibles. Euplerids overlap with all extant feliform families in mandibular shape morphospace, whereas they only overlap with non-hyercarnivorous families (i.e. Herpestidae, Nandiniidae, Prionodontidae and Viverridae) in cranial shape morphospace (figure 2). The only euplerid to approach hypercarnivorous feliforms (Felidae, Hyaenidae) in both cranial and mandibular morphospace is the fossa (*C. ferox*), the most carnivorous of the euplerids [22]. The fossa exhibits cranial and mandibular morphologies that appear intermediate between the average euplerid and average felid (figures 5*a* and 6*a*). Compared to all other euplerids, the fossa exhibits relatively broader zygomatic arches and more pronounced sagittal and nuchal crests in the cranium (figure 5*a*) and relatively broader coronoid processes in the mandible (figure 6*a*). These adaptations suggest that the fossa exhibit larger temporalis and masseter jaw muscles for increased biting ability [64–67] and are thus better adapted for their hypercarnivorous diets [22]. There is some evidence that the fossa and felids share a similar adaptive zone in cranial shape (∆AIC = 1.47; electronic supplementary material, table S2), but there is no statistical support for convergence in skull shape between the fossa and felids. These results suggest that cranial adaptations towards hypercarnivory in the fossa do not quite reach the felid morphotype (figures 5*a* and 6*a*) because the fossa and felids share an adaptive zone characterized by broad adaptive slopes rather than distinct adaptive peaks with steep slopes [24,52,68,69].

Euplerines occupy overlapping regions of cranial and mandibular morphospace with viverrids, the family to which they have been compared or classified with historically (figure 2). The fanaloka (*F. fossana*) exhibits only slight differences in skull shape (i.e. narrower and more elongate crania and mandibles) compared to the average viverrid, which is consistent with their similar diets of invertebrate and vertebrate prey, whereas the falanouc (*E. goudotii*) exhibits a more specialized skull that includes relatively elongate rostrum and mandible and less pronounced zygomatic arches, nuchal crest, sagittal crests, coronoid processes compared to the average viverrid (figure 5*b*) and all other feliforms. These adaptations in the skull, along with reduced dentition, enable the falanouc to specialize in feeding on soft-bodied invertebrates such as worms and slugs [22]. We found evidence that euplerines and viverrids share an adaptive zone in cranial shape but not mandibular shape (electronic supplementary material, table S2), but neither skull component provided statistical support for convergence between the two groups. Therefore, like for the fossa and felids, our results suggest that euplerines and viverrids may share an adaptive zone with broad adaptive slopes instead of exhibiting convergence characterized by distinct similar adaptive peaks with steep slopes.

Galidiines exhibit distinct patterns of cranial and mandibular shape distribution in morphospace. Galidiines occupy overlapping regions of cranial morphospace with herpestids (figure 2), and there appear to be few cranial shape differences between the galidiine species closest to the average cranial shape of all galidiines and the herpestid species closest to the average cranial shape of all herpestids (figure 5*c*). Evolutionary modelling also strongly supports a shared adaptive zone in cranial shape between galidiines and herpestids (electronic supplementary material, table S2) but no statistical support for convergence. However, galidiines approach regions of mandibular morphospace occupied by hypercarnivorous feliforms (figure 2) and exhibit mandibular morphologies that appear intermediate between herpestids and felids (figure 6*c*,*d*): galidiine mandibles tend to be relatively broader and more robust compared to the herpestid species closest to the average mandibular shape of all herpestids but are not as broad or robust as the felid species closest to the average mandibular shape of all felids (figure 6*c*,*d*). Of the galidiines, *Galidictis* species appear most similar to felids in mandibular shape, providing morphological evidence that carnivory likely is an important component of their diet. Previous work surmised that, in addition to insects, *Galidictis* feed on rodents, reptiles, amphibians and even small lemurs; however, their dietary ecologies remain poorly known, as they are some of the rarest carnivorans in the world [22]. That *Galidictis* exhibits a rate shift towards faster mandibular evolution (figure 3) leads us to postulate that shifts towards more carnivorous diets may be due to more relatively recent (<5 million years ago) selection. The distinct patterns between the cranium and mandible in galidiines follow an overall trend of decoupled modes of evolution between the cranium and mandible across carnivorans, where cranial evolution tends to follow clade-based shifts, whereas mandibular evolution is linked instead to broad dietary regimes [36,70]. Our results suggest that galidiines may have retained cranial morphology from euplerid–herpestid ancestors through conservatism rather than convergence, whereas the more evolutionary labile mandible adapted towards a more carnivorous diet than the typical insectivorous diets found in herpestids, most euplerids, and most likely their most recent common ancestors (of the euplerid + herpestid clade).

### 4.2. Are Malagasy carnivorans an adaptive radiation?

Like many endemic Malagasy clades, euplerids are often considered to represent an adaptive radiation after dispersal to a long-isolated island. We found that euplerids show extreme ranges of skull shape variation and many distinct cranial and mandibular shapes in phylomorphospace and geometric morphometric analyses. Contrary to predictions of adaptive radiation, we found that euplerids do not exhibit exceptional lineage diversification and have only marginally faster rates of mandibular shape evolution and, to a lesser extent, cranial shape evolution compared to other feliform families (figures 3 and 4). Thus, it is tempting to postulate that euplerids may have evolved via an ‘adaptive non-radiation’ [8], where ecomorphological divergence within a clade fails to produce rapid species diversification. Rapid phenotypic evolution without rapid taxonomic diversification rates has been found in a variety of vertebrate clades [71–73] including other carnivorans such as mustelids [74] as well as other endemic Malagasy vertebrates such as mantellid frogs [8]. Those studies cited possible factors that may mask a signal of rapid diversification [8,71–74] including the absence of fossil data in diversification analyses, which may have erased signatures of early rapid diversification [75–77]; continental radiations, in which many clades are simply unable to rapidly radiate spatially across entire continents as soon as they arise [72,74,78–80]; and clade age, where younger clades diversify faster than older clades [81]. These factors also may influence our analyses of euplerid diversification. First, euplerids are not an exceptionally young clade, having diverged from herpestids ~24−18 million years ago [30] with a clade age for extant species of ~15−14 million years old [33]. Despite their long evolutionary history, euplerids are extremely poorly represented in the fossil record, known only from Holocene subfossil remains of the extinct giant fossa (*C. spelea*) [82]. Therefore, unaccounted cladogenesis of extinct euplerids may lead to underestimation of taxonomic diversification rates and ecological diversity in euplerids. Second, unaccounted speciation of modern euplerids also may lead to underestimation of diversification rates. At least two euplerids—*E. goudotii* and *S. concolor*—are thought, though with uncertainty, to each consist of two species (i.e. *E. goudotii* and *E. major* and *S. concolor* and *S. durrelli*) [41,42,83]; if valid, this would lead to increased diversification rates, albeit at more crownward tips of the tree. Lastly, euplerid diversification is perhaps better characterized as a continental radiation rather than as an insular radiation. Researchers often suggest that Madagascar, as the fourth largest island globally, resembles a continent more than an island, with a geological record that spans more than three billion years and several vicariance and dispersal events over the past ~88–90 million years, after its final separation from other major Gondwanan landmasses [84–86]. Therefore, the diversification of Malagasy clades with long residence times in Madagascar may be better characterized as multiple bursts of speciation events throughout their evolutionary history [19,87] rather than as a single early burst of rapid diversification that has been found in some other insular clades [9–11]. That diversification rates do not differ statistically between euplerids and continental feliforms support the hypothesis that the diversification dynamics of other endemic Malagasy clades follow a pattern more similar to diversification on a continent than on an island [8]. Therefore, while found only on Madagascar, there is no indication that insular processes spurred a rapid burst of taxonomic diversification of euplerids, as often has been hypothesized.

## 5. Conclusion

Euplerids exhibit great disparity in cranial and mandibular morphology that corresponds to their wide dietary diversity ranging from worm-eating falanoucs to lemur-eating fossas. We document for the first time that their skull morphologies, particularly their mandibles, show significantly greater shape disparity compared to other feliform clades, and this single small clade of Malagasy endemic carnivorans captures a large amount of the morphological variation that is found across the entire clade of feliforms. We also found evidence that the three euplerid clades—galidiines, euplerines, and the fossa—exhibited shared adaptive zones in cranial shape morphology with herpestids, viverrids and felids, respectively. We did not find statistical evidence, however, that these three euplerid clades—galidiines, euplerines and the fossa—converged towards herpestid-, viverrid- and felid-morphotypes in skull morphology, respectively, as had been implicit in classifications or directly hypothesized historically. Despite demonstrating great skull disparity, we found that euplerids do not clearly exhibit an adaptive radiation, as we found no evidence of rapid lineage diversification rates and only marginally faster rates of mandibular shape evolution compared to other feliform families. This work supports an increasing number of studies demonstrating that adaptive radiations in endemic Malagasy clades may not as common as previously thought [8,19] and diversification dynamics on Madagascar may resemble those found on continents rather than on islands.

## Data Availability

All data and original code are available from the Dryad Digital Repository [88]. Supplementary material is available online [89].

## References

[1] Myers N, Mittermeier RA, Mittermeier CG, da Fonseca GAB, Kent J. 2000 Biodiversity hotspots for conservation priorities. Nature **403**, 853–858. (10.1038/35002501) 10706275

[2] Kier G, Kreft H, Lee TM, Jetz W, Ibisch PL, Nowicki C, Mutke J, Barthlott W. 2009 A global assessment of endemism and species richness across island and mainland regions. Proc. Natl Acad. Sci. USA **106**, 9322–9327. (10.1073/pnas.0810306106) 19470638 PMC2685248

[3] Losos JB, Ricklefs RE. 2009 Adaptation and diversification on islands. Nature **457**, 830–836. (10.1038/nature07893) 19212401

[4] Veron S, Haevermans T, Govaerts R, Mouchet M, Pellens R. 2019 Distribution and relative age of endemism across islands worldwide. Sci. Rep. **9**, 11693. (10.1038/s41598-019-47951-6) 31406123 PMC6690940

[5] Stroud JT, Losos JB. 2016 Ecological opportunity and adaptive radiation. Annu. Rev. Ecol. Evol. Syst. **47**, 507–532. (10.1146/annurev-ecolsys-121415-032254)

[6] Simpson GG. 1955 Major features of evolution. New York, NY: Columbia University Press. See http://krishikosh.egranth.ac.in/handle/1/2034793.

[7] Schluter D. 2000 The ecology of adaptive radiation. Oxford, UK: Oxford University Press. See http://books.google.com/books/content?id=Q1wxNmLAL10C&printsec=frontcover&img=1&zoom=1&edge=curl&source=gbs_api.

[8] Moen DS, Ravelojaona RN, Hutter CR, Wiens JJ. 2021 Testing for adaptive radiation: a new approach applied to Madagascar frogs. Evolution **75**, 3008–3025. (10.1111/evo.14328) 34396527

[9] Grant PR, Grant BR. 2007 How and why species multiply: the radiation of Darwin’s finches. Princeton, NJ: Princeton University Press.

[10] Losos JB. 2011 Lizards in an evolutionary tree: ecology and adaptive radiation of anoles. Berkeley, CA: University of California Press.

[11] Lerner HRL, Meyer M, James HF, Hofreiter M, Fleischer RC. 2011 Multilocus resolution of phylogeny and timescale in the extant adaptive radiation of Hawaiian honeycreepers. Curr. Biol. **21**, 1838–1844. (10.1016/j.cub.2011.09.039) 22018543

[12] Ralimanana H *et al*. 2022 Madagascar’s extraordinary biodiversity: threats and opportunities. Science **378**, eadf1466. (10.1126/science.adf1466) 36454830

[13] Yoder AD, Nowak MD. 2006 Has vicariance or dispersal been the predominant biogeographic force in Madagascar? Only time will tell. Annu. Rev. Ecol. Evol. Syst. **37**, 405–431. (10.1146/annurev.ecolsys.37.091305.110239)

[14] Wirta H, Orsini L, Hanski I. 2008 An old adaptive radiation of forest dung beetles in Madagascar. Mol. Phylogenet. Evol. **47**, 1076–1089. (10.1016/j.ympev.2008.03.010) 18424187

[15] Moore W, Robertson JA. 2014 Explosive adaptive radiation and extreme phenotypic diversity within ant-nest beetles. Curr. Biol. **24**, 2435–2439. (10.1016/j.cub.2014.09.022) 25283783

[16] Wollenberg Valero KC *et al*. 2017 Transcriptomic and macroevolutionary evidence for phenotypic uncoupling between frog life history phases. Nat. Commun. **8**, 15213. (10.1038/ncomms15213) 28504275 PMC5440664

[17] Bossuyt F, Milinkovitch MC. 2000 Convergent adaptive radiations in Madagascan and Asian ranid frogs reveal covariation between larval and adult traits. Proc. Natl Acad. Sci. USA **97**, 6585–6590. (10.1073/pnas.97.12.6585) 10841558 PMC18667

[18] Reddy S, Driskell A, Rabosky DL, Hackett SJ, Schulenberg TS. 2012 Diversification and the adaptive radiation of the vangas of Madagascar. Proc. R. Soc. B **279**, 2062–2071. (10.1098/rspb.2011.2380) PMC3311898 22217720

[19] Everson KM *et al*. 2023 Not one, but multiple radiations underlie the biodiversity of Madagascar’s endangered lemurs. bioRxiv. (10.1101/2023.04.26.537867)

[20] Godfrey LR, Samonds KE, Baldwin JW, Sutherland MR, Kamilar JM, Allfisher KL. 2020 Mid-cenozoic climate change, extinction, and faunal turnover in Madagascar, and their bearing on the evolution of lemurs. BMC Evol. Biol. **20**, 97. (10.1186/s12862-020-01628-1) 32770933 PMC7414565

[21] Herrera JP. 2016 Testing the adaptive radiation hypothesis for the lemurs of Madagascar. R. Soc. Open Sci. **4**, 161014. (10.1098/rsos.161014) PMC5319363 28280597

[22] Goodman SM. 2009 Family Eupleridae. Madagascar carnivores. In Handbook of the mammals of the world, volume 1, carnivores (eds D Wilson, RA Mittermeier), pp. 330–351. Barcelona, Spain: Lynx Edicions.

[23] Michaud M, Veron G, Peigné S, Blin A, Fabre A-C. 2018 Are phenotypic disparity and rate of morphological evolution correlated with ecological diversity in Carnivora? Biol. J. Linnean Soc. **124**, 294–307. (10.1093/biolinnean/bly047/4994739)

[24] Law CJ. 2022 Different evolutionary pathways lead to incomplete convergence of elongate body shapes in carnivoran mammals. Syst. Biol. **71**, 788–796. (10.1093/sysbio/syab091) 34791502

[25] Law CJ. 2021 Evolutionary and morphological patterns underlying carnivoran body shape diversity. Evolution **75**, 365–375. (10.1111/evo.14143) 33314085

[26] Lydekker R. 1894 A hand-book to the Carnivora. Part 1. Cats, civets, and mungooses. London, UK: Allen. (10.5962/bhl.title.9520)

[27] Pocock RI. 1915 XLIV.—On some external characters of Galidia, Galidictis, and related genera. J. Nat. Hist. Ser. 8 16, 351–356. (10.1080/00222931508693726)

[28] Gregory WK, Hellman M. 1939 On the evolution and major classification of the civets (Viverridae) and allied fossil and recent Carnivora: a phylogenetic study of the skull and dentition. Proc. Am. Philos. Soc. 309–392.

[29] Bennett ET. 1833 Notice of a new genus of viverridous Mammalia from Madagascar. Proc. Zool. Soc. Lond. **Pt I**, 46.

[30] Yoder AD, Burns MM, Zehr S, Delefosse T, Veron G, Goodman SM, Flynn JJ. 2003 Single origin of Malagasy Carnivora from an African ancestor. Nature **421**, 734–737. (10.1038/nature01303) 12610623

[31] Yoder AD, Flynn JJ. 2003 Origin of Malagasy Carnivora. In The natural history of Madagascar, pp. 1253–1256. Chicago, IL: University of Chicago Press.

[32] Flynn JJ, Finarelli JA, Zehr S, Hsu J, Nedbal MA. 2005 Molecular phylogeny of the Carnivora (Mammalia): assessing the impact of increased sampling on resolving enigmatic relationships. Syst. Biol. **54**, 317–337. (10.1080/10635150590923326) 16012099

[33] Upham NS, Esselstyn JA, Jetz W. 2019 Inferring the mammal tree: species-level sets of phylogenies for questions in ecology, evolution, and conservation. PLoS Biol. **17**, e3000494. (10.1371/journal.pbio.3000494) 31800571 PMC6892540

[34] Wilson DE, Mittermeier RA. 2009 Handbook of the mammals of the world, volume 1, carnivores. Barcelona, Spain: Lynx Edicions. See http://www.sidalc.net/cgi-bin/wxis.exe/?IsisScript=sibe01.xis&method=post&formato=2&cantidad=1&expresion=mfn=034105.

[35] Law CJ, Duran E, Hung N, Richards E, Santillan I, Mehta RS. 2018 Effects of diet on cranial morphology and biting ability in musteloid mammals. J. Evol. Biol. **31**, 1918–1931. (10.1111/jeb.13385) 30270461

[36] Law CJ, Blackwell EA, Curtis AA, Dickinson E, Hartstone-Rose A, Santana SE. 2022 Decoupled evolution of the cranium and mandible in carnivoran mammals. Evolution **76**, 2959–2974. (10.1111/evo.14578) 35875871

[37] Figueirido B, Tseng ZJ, Martín-Serra A. 2013 Skull shape evolution in durophagous carnivorans. Evolution **67**, 1975–1993. (10.1111/evo.12059) 23815654

[38] Meloro C, Clauss M, Raia P. 2015 Ecomorphology of Carnivora challenges convergent evolution. Org. Divers. Evol. **15**, 1–10. (10.1007/s13127-015-0227-5)

[39] Rohlf FJ, Slice D. 1990 Extensions of the procrustes method for the optimal superimposition of landmarks. Syst. Zool. **39**, 21–40. (10.2307/2992207)

[40] Zelditch ML, Swiderski DL, Sheets HD. 2012 Geometric morphometrics for biologists: a primer. San Diego, CA: Academic Press.

[41] Veron G, Goodman SM. 2018 One or two species of the rare Malagasy carnivoran Eupleres (Eupleridae)? New insights from molecular data. Mammalia **82**, 107–112. (10.1515/mammalia-2016-0182)

[42] Durbin J, Funk SM, Hawkins F, Hills DM, Jenkins PD, Moncrieff CB, Ralainasolo FB. 2010 Investigations into the status of a new taxon of Salanoia (Mammalia: Carnivora: Eupleridae) from the marshes of Lac Alaotra, Madagascar. Syst. Biodivers. **8**, 341–355. (10.1080/14772001003756751)

[43] Law CJ. 2019 Solitary meat-eaters: solitary, carnivorous carnivorans exhibit the highest degree of sexual size dimorphism. Sci. Rep. **9**, 15344. (10.1038/s41598-019-51943-x) 31653949 PMC6814822

[44] Law CJ, Mehta RS. 2018 Carnivory maintains cranial dimorphism between males and females: evidence for niche divergence in extant Musteloidea. Evolution **72**, 1950–1961. (10.1111/evo.13514) 29882586

[45] Bookstein FL. 1997 Landmark methods for forms without landmarks: morphometrics of group differences in outline shape. Med. Image Anal. **1**, 225–243. (10.1016/s1361-8415(97)85012-8) 9873908

[46] Baken EK, Collyer ML, Kaliontzopoulou A, Adams DC. 2021 geomorph v4.0 and gmShiny: enhanced analytics and a new graphical interface for a comprehensive morphometric experience. Methods Ecol. Evol. **12**, 2355–2363. (10.1111/2041-210X.13723)

[47] R Core Team. 2023 R: a language and environment for statistical computing. Vienna, Austria: R Foundation for Statistical Computing. See https://www.R-project.org/.

[48] Mitchell DR, Sherratt E, Weisbecker V. 2023 Facing the facts: adaptive trade-offs along body size ranges determine mammalian craniofacial scaling. Biol. Rev. **99**, 496–524. (10.1111/brv.13032) 38029779

[49] Adams DC. 2014 A method for assessing phylogenetic least squares models for shape and other high-dimensional multivariate data. Evolution **68**, 2675–2688. (10.1111/evo.12463) 24899536

[50] Losos JB. 2011 Convergence, adaptation, and constraint. Evolution **65**, 1827–1840. (10.1111/j.1558-5646.2011.01289.x) 21729041

[51] Stayton CT. 2015 The definition, recognition, and interpretation of convergent evolution, and two new measures for quantifying and assessing the significance of convergence. Evolution **69**, 2140–2153. (10.1111/evo.12729) 26177938

[52] Grossnickle DM, Brightly WH, Weaver LN, Stanchak KE, Roston RA, Pevsner SK, Stayton CT, Polly PD, Law CJ. 2024 Challenges and advances in measuring phenotypic convergence. Evolution **78**, 1355–1371. (10.1093/evolut/qpae081) 38771219

[53] Castiglione S *et al*. 2019 A new, fast method to search for morphological convergence with shape data. PLoS ONE **14**, e0226949. (10.1371/journal.pone.0226949) 31881075 PMC6934287

[54] Hansen TF. 1997 Stabilizing selection and the comparative analysis of adaptation. Evolution **51**, 1341–1351. (10.1111/j.1558-5646.1997.tb01457.x) 28568616

[55] Butler MA, King AA. 2004 Phylogenetic comparative analysis: a modeling approach for adaptive evolution. Am. Nat. **164**, 683–695. (10.1086/426002) 29641928

[56] Clavel J, Escarguel G, Merceron G. 2015 mv morph: an R package for fitting multivariate evolutionary models to morphometric data. Methods Ecol. Evol. **6**, 1311–1319. (10.1111/2041-210X.12420)

[57] Coombs EJ, Felice RN, Clavel J, Park T, Bennion RF, Churchill M, Geisler JH, Beatty B, Goswami A. 2022 The tempo of cetacean cranial evolution. Curr. Biol. **32**, 2233–2247.e4. (10.1016/j.cub.2022.04.060) 35537454

[58] Rabosky DL. 2014 Automatic detection of key innovations, rate shifts, and diversity-dependence on phylogenetic trees. PLoS ONE **9**, e89543-15. (10.1371/journal.pone.0089543) 24586858 PMC3935878

[59] Höhna S, Freyman WA, Nolen Z, Huelsenbeck JP, May MR, Moore BR. 2019 A Bayesian approach for estimating branch-specific speciation and extinction rates. bioRxiv. (10.1101/555805)

[60] Rabosky DL, Grundler M, Anderson C, Title P, Shi JJ, Brown JW, Huang H, Larson JG. 2014 BAMMtools: an R package for the analysis of evolutionary dynamics on phylogenetic trees. Methods Ecol. Evol. **5**, 701–707. (10.1111/2041-210X.12199)

[61] Höhna S, Landis MJ, Heath TA, Boussau B, Lartillot N, Moore BR, Huelsenbeck JP, Ronquist F. 2016 RevBayes: Bayesian phylogenetic inference using graphical models and an interactive model-specification language. Syst. Biol. **65**, 726–736. (10.1093/sysbio/syw021) 27235697 PMC4911942

[62] Tribble CM, Freyman WA, Landis MJ, Lim JY, Barido‐Sottani J, Kopperud BT, Hӧhna S, May MR. 2022 RevGadgets: an R package for visualizing Bayesian phylogenetic analyses from RevBayes. Methods Ecol. Evol. **13**, 314–323. (10.1111/2041-210X.13750)

[63] Plummer M, Best N, Cowles K, Vines K. 2006 CODA: convergence diagnosis and output analysis for MCMC. R News **6**, 7–11.

[64] Radinsky LB. 1981 Evolution of skull shape in carnivores: 1. Representative modern carnivores. Biol. J. Linnean Soc. **15**, 369–388. (10.1111/j.1095-8312.1981.tb00770.x)

[65] Law CJ, Young C, Mehta RS. 2016 Ontogenetic scaling of theoretical bite force in southern sea otters (Enhydra lutris nereis). Physiol. Biochem. Zool. **89**, 347–363. (10.1086/688313) 27617357

[66] Dickinson E *et al*. 2021 Evaluating bony predictors of bite force across the order Carnivora. J. Morphol. **282**, 1499–1513. (10.1002/jmor.21400) 34313337

[67] Hartstone‐Rose A, Dickinson E, Deutsch AR, Worden N, Hirschkorn GA. 2021 Masticatory muscle architectural correlates of dietary diversity in Canidae, Ursidae, and across the order Carnivora. Anat. Rec. **305**, 477–497. (10.1002/ar.24748) 34449131

[68] Phillips PC, Arnold SJ. 1989 Visualizing multivariate selection. Evolution **43**, 1209–1222. (10.1111/j.1558-5646.1989.tb02569.x) 28564514

[69] Polly PD. 2004 On the simulation of the evolution of morphological shape: multivariate shape under selection and drift. Palaeontol. Electron. **7**, 7.2.7A.

[70] Law CJ, Hlusko LJ, Tseng ZJ. 2024 Uncovering the mosaic evolution of the carnivoran skeletal system. Biol. Lett. **20**, 20230526. (10.1098/rsbl.2023.0526) 38263882 PMC10806395

[71] Adams DC, Berns CM, Kozak KH, Wiens JJ. 2009 Are rates of species diversification correlated with rates of morphological evolution? Proc. R. Soc. B **276**, 2729–2738. (10.1098/rspb.2009.0543) PMC2839962 19439441

[72] Derryberry EP, Claramunt S, Derryberry G, Chesser RT, Cracraft J, Aleixo A, Pérez-Emán J, Remsen Jr. JV, Brumfield RT. 2011 Lineage diversification and morphological evolution in a large-scale continental radiation: the neotropical ovenbirds and woodcreepers (Aves: Furnariidae). Evolution **65**, 2973–2986. (10.1111/j.1558-5646.2011.01374.x) 21967436

[73] Aristide L, Rosenberger AL, Tejedor MF, Perez SI. 2015 Modeling lineage and phenotypic diversification in the New World monkey (Platyrrhini, Primates) radiation. Mol. Phylogenet. Evol. **82**, 375–385. (10.1016/j.ympev.2013.11.008) 24287474

[74] Law CJ, Slater GJ, Mehta RS. 2018 Lineage diversity and size disparity in Musteloidea: testing patterns of adaptive radiation using molecular and fossil-based methods. Syst. Biol. **67**, 127–144. (10.1093/sysbio/syx047) 28472434

[75] Rabosky DL, Lovette IJ. 2008 Explosive evolutionary radiations: decreasing speciation or increasing extinction through time? Evolution **62**, 1866–1875. (10.1111/j.1558-5646.2008.00409.x) 18452577

[76] Quental TB, Marshall CR. 2009 Extinction during evolutionary radiations: reconciling the fossil record with molecular phylogenies. Evolution **63**, 3158–3167. (10.1111/j.1558-5646.2009.00794.x) 19659595

[77] Liow LH, Quental TB, Marshall CR. 2010 When can decreasing diversification rates be detected with molecular phylogenies and the fossil record? Syst. Biol. **59**, 646–659. (10.1093/sysbio/syq052) 20861283

[78] Day JJ, Peart CR, Brown KJ, Friel JP, Bills R, Moritz T. 2013 Continental diversification of an African catfish radiation (Mochokidae: Synodontis). Syst. Biol. **62**, 351–365. (10.1093/sysbio/syt001) 23302956

[79] Kozak KM, Wahlberg N, Neild AFE, Dasmahapatra KK, Mallet J, Jiggins CD. 2015 Multilocus species trees show the recent adaptive radiation of the mimetic heliconius butterflies. Syst. Biol. **64**, 505–524. (10.1093/sysbio/syv007) 25634098 PMC4395847

[80] Liedtke HC *et al*. 2016 No ecological opportunity signal on a continental scale? Diversification and life-history evolution of African true toads (Anura: Bufonidae). Evolution **70**, 1717–1733. (10.1111/evo.12985) 27312525

[81] Henao Diaz LF, Harmon LJ, Sugawara MTC, Miller ET, Pennell MW. 2019 Macroevolutionary diversification rates show time dependency. Proc. Natl Acad. Sci. USA **116**, 7403–7408. (10.1073/pnas.1818058116) 30910958 PMC6462100

[82] Goodman SM, Rasoloarison RM, Ganzhorn JU. 2004 On the specific identification of subfossil Cryptoprocta (Mammalia, Carnivora) from Madagascar. Zoosystema **26**, 129–143.

[83] Goodman SM, Helgen KM. 2010 Species limits and distribution of the Malagasy carnivoran genus Eupleres (Family Eupleridae). Mammalia **74**, 177–185. (10.1515/mamm.2010.018)

[84] Wit M de. 2003 Madagascar: heads it’s a continent, tails it’s an island. Annu. Rev. Earth Planet. Sci. **31**, 213–248. (10.1146/annurev.earth.31.100901.141337)

[85] Vences M, Wollenberg KC, Vieites DR, Lees DC. 2009 Madagascar as a model region of species diversification. Trends Ecol. Evol. **24**, 456–465. (10.1016/j.tree.2009.03.011) 19500874

[86] Krause DW, O’Connor PM, Sertich JJW, Rogers KC, Rogers RR, Rakotozafy B. 2022 Late Cretaceous vertebrates of Madagascar: a window into Gondwanan biogeography. In The new natural history of Madagascar, pp. 59–68. Princeton, NJ: Princeton University Press. (10.2307/j.ctv2ks6tbb.13)

[87] Raxworthy CJ, Pearson RG, Zimkus BM, Reddy S, Deo AJ, Nussbaum RA, Ingram CM. 2008 Continental speciation in the tropics: contrasting biogeographic patterns of divergence in the Uroplatus leaf‐tailed gecko radiation of Madagascar. J. Zoöl. **275**, 423–440. (10.1111/j.1469-7998.2008.00460.x)

[88] Law CJ, Linden TJ, Flynn JJ. 2024 Skull evolution and lineage diversification in endemic Malagasy carnivorans. Dryad Digital Repository. (10.5061/dryad.x95x69psj) PMC11496717 39445090

[89] Law CJ, Linden T, Flynn J. 2024 Supplementary material from: Skull evolution and lineage diversification in endemic Malagasy carnivorans. FigShare (10.6084/m9.figshare.c.7502588) PMC11496717 39445090

